# Chemotherapeutic Stress Influences Epithelial–Mesenchymal Transition and Stemness in Cancer Stem Cells of Triple-Negative Breast Cancer

**DOI:** 10.3390/ijms21020404

**Published:** 2020-01-08

**Authors:** Xiao Li, Juliane Strietz, Andreas Bleilevens, Elmar Stickeler, Jochen Maurer

**Affiliations:** 1 Department of Obstetrics and Gynecology, University Hospital Aachen (UKA), 52074 Aachen, Germany; xli@ukaachen.de (X.L.); ableilevens@ukaachen.de (A.B.); estickeler@ukaachen.de (E.S.); 2 Department of Immunology, University of Freiburg, 79104 Freiburg, Germany; juliane.strietz@biologie.uni-freiburg.de

**Keywords:** breast cancer stem cell, chemotherapeutic stress, EMT, stemness, miRNA

## Abstract

Triple-negative breast cancer (TNBC) is a subtype of breast cancer characterized by the absence of estrogen and progesterone receptors (ER, PR) and lacking an overexpression of human epidermal growth factor receptor 2 (HER2). Apart from this lack of therapeutic targets, TNBC also shows an increased capacity for early metastasis and therapy resistance. Currently, many TNBC patients receive neoadjuvant chemotherapy (NACT) upon detection of the disease. With TNBC likely being driven at least in part by a cancer stem-like cell type, we wanted to evaluate the response of primary cancer stem cells (CSCs) to standard chemotherapeutics. Therefore, we set up a survival model using primary CSCs to mimic tumor cells in patients under chemotherapy. Breast cancer stem cells (BCSCs) were exposed to chemotherapeutics with a sublethal dose for six days. Surviving cells were allowed to recover in culture medium without chemotherapeutics. Surviving and recovered cells were examined in regard to proliferation, migratory capacity, sphere forming capacity, epithelial–mesenchymal transition (EMT) factor expression at the mRNA level, and cancer-related microRNA (miRNA) profile. Our results indicate that chemotherapeutic stress enhanced sphere forming capacity of BCSCs, and changed cell morphology and EMT-related gene expression at the mRNA level, whereas the migratory capacity was unaffected. Six miRNAs were identified as potential regulators in this process.

## 1. Introduction

Breast cancer is the most commonly diagnosed cancer and the major cause of cancer-related mortality in women worldwide [1]. Among different subtypes of breast cancer, TNBC is an aggressive, poor prognosis subtype which accounts for approximately 12–17% of breast cancer cases [2]. The treatment regimens currently adopted for TNBC are mostly based on an anthracycline and taxane backbone and are frequently applied in a neoadjuvant, preoperative setting [3]. Although it is accepted that early-stage TNBC is chemosensitive, high recurrence rates, early metastasis, and development of therapy resistance are major challenges bringing this breast cancer subtype into a major focus of current translational and clinical research efforts in order to develop innovative treatment options to improve clinical patient outcome.

Over the last decade, the CSC hypothesis has been proposed as a mechanism underlying both cancer metastasis and therapy resistance [4]. The CSC phenotype is usually not rigid but highly dynamic, which could be linked to an intermediate EMT state [5]. This partial EMT state could be advantageous for metastasis because the cells combine stemness and invasiveness in one state [6]. In order to elucidate the mechanism of metastasis and therapy resistance in TNBC, the regulation of CSC phenotype and EMT state should be defined first.

TNBC seems to be at least in part driven by cancer stem cells. Several studies reported CSC markers to be higher expressed in TNBC than in other intrinsic subtypes [7,8]. Furthermore, TNBC cells show a higher degree of self-renewal [9] and upregulate several pluripotency-related factors, such as Myc and Sox2 [10,11].

MiRNAs are non-coding RNA molecules, which play important roles in physiological and pathological processes by post-transcriptional regulation of gene expression [12]. In recent years, numerous studies revealed the function of miRNAs as potential biomarkers and key modulators in breast cancer [13,14]. CSCs are at least in part responsible for chemoresistance in tumors [4,15], the chemotherapeutic-dependent regulation of miRNAs in BCSCs are yet unknown.

In the present study, we examine the effect of sublethal doses of common chemotherapeutics on primary BCSCs from TNBC patients. With the use of standard chemotherapeutics such as doxorubicin and paclitaxel we model how BCSCs react to chemotherapeutic stress in vitro and draw conclusions for their behavior in vivo. Sublethal doses of chemotherapeutics change the cellular morphology of BCSCs and alter EMT-related gene expression, but not migratory capacity. Furthermore, we find enhanced self-renewal capacity of BCSCs after chemotherapeutic stress. MiRNA profiles are examined to define regulatory pathways and biomarkers for a chemoresistant phenotype.

## 2. Results

### 2.1. BCSCs from TNBC Show Varying Resistance Against Different Chemotherapeutics

**Figure 1:**
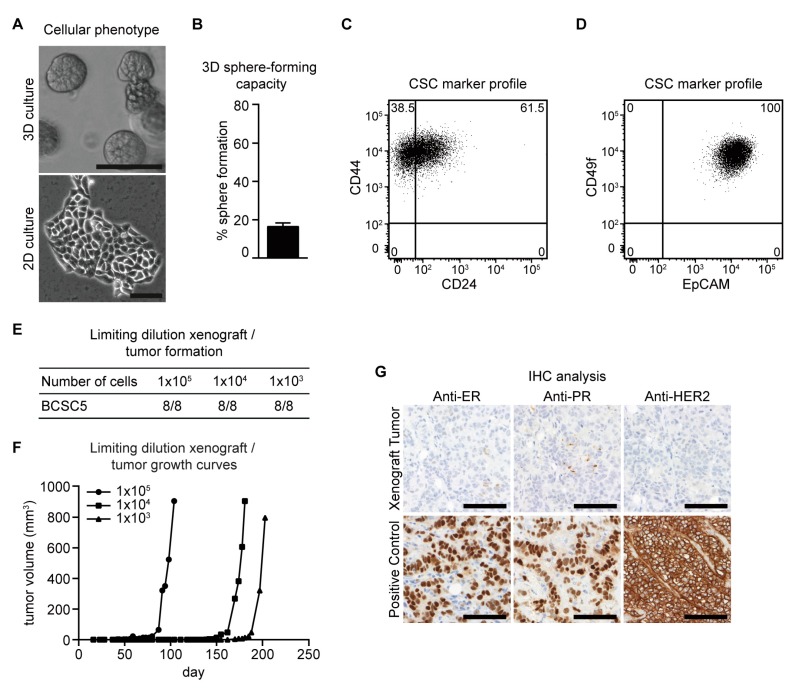
Characterization of BCSC5 in vitro and in vivo. (**A**) Representative pictures of BCSC5 cultured in 3D and 2D conditions, scale bar 100 μm. (**B**) Sphere-forming capacity of BCSC5 cells in an anchorage-independent growth assay (*n* = 3). Data represent means + SEM. (**C**,**D**) Expression patterns of CD24 and CD44 (**C**) as well as EpCAM and CD49f (**D**) in BCSC5 cells analyzed by flow cytometry. (**E**) Tumor formation in limiting dilution xenografts of BCSC5. (**F**) Representative growth curves for limiting dilution assay of BCSC5 xenografts in immunocompromised NOD/SCID mice. (**G**) Immunohistochemical (IHC) analysis of ER, PR, and HER2 on sections of the BCSC5 xenograft tumors, scale bar 100 μm.

In a previously published study, we established several breast cancer stem cell lines isolated from primary invasive breast cancer of individual patients [16]. All patients were diagnosed with TNBC and treated with different regimens of NACT. Specimens were taken during surgical treatment after exposure to chemotherapy. As shown in Figure 1, we established and evaluated BCSC5 with the same methods used for BCSC1 and BCSC2 described in our previous study [16]. BCSC5 could be cultivated in both 3D and 2D cell culture conditions (Figure 1A). BCSC5 had a colony-forming capacity of 16.4% when tested in an anchorage-independent growth assay (Figure 1B). Surface marker analysis showed 38.5% CD24^−^/CD44^+^ phenotype and 100% EpCAM^+^/CD49f^+^ cells in BCSC5 (Figure 1C,D). Limiting dilution orthotopic xenografts in immunocompromised NOD/SCID mice demonstrated that BCSC5 exhibited a high tumorigenic potential with tumors forming from as few as 1000 cells (Figure 1E,F). Immunohistochemical analyses of BCSC5 xenograft tumors confirmed the classification as triple-negative (Figure 1G). Taken together, all the cell lines used in this study showed triple-negative characteristics and were characterized in multiple assays as BCSCs.

As previously described, the patient BCSC1 was isolated from was uncommonly preoperatively treated with sequential chemotherapy regimens, including FEC (5-fluorouracil, epirubicin, and cyclophosphamide), FAC (5-fluorouracil, doxorubicin, and cyclophosphamide), TAC (docetaxel, doxorubicin, and cyclophosphamide), TC (docetaxel and cyclophosphamide), and cisplatin. This cell line was chosen as an example for heavily pretreated tumors after several lines of chemotherapy, potentially reflecting the clinical course of disease after first recurrence and sequential treatment lines after further progression.

In contrast, the patient of the BCSC2 cell line was treated with neoadjuvant paclitaxel and doxorubicin, representing the situation if a complete pathological remission after NACT is not achieved indicating a high risk for distant tumor recurrence within the next three years [16]. Although the patient of BCSC5 was pretreated with NACT, information on the chemotherapy regimen was incomplete.

**Figure 2:**
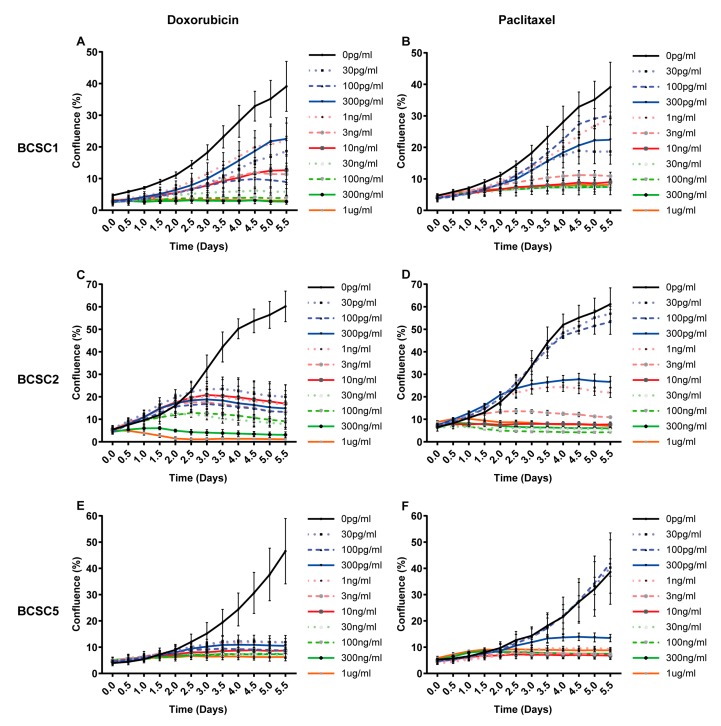
BCSCs from TNBC show different resistance against doxorubicin and paclitaxel. BCSCs were treated with doxorubicin and paclitaxel at 10 different concentrations ranging from 30 pg/mL to 1 μg/mL. Cell confluence was recorded and analyzed using automated phase contrast microscopy. (**A**) Dose response curves over time for BCSC1 under doxorubicin, (**B**) BCSC1 under paclitaxel (**C**) BCSC2 under doxorubicin (**D**) BCSC2 under paclitaxel, (**E**) BCSC5 under doxorubicin, (**F**) BCSC5 under paclitaxel.

Since BCSCs were isolated from tumors after chemotherapy, we hypothesized these cells should display a certain degree of chemoresistance. To address this hypothesis, eight commonly used chemotherapeutics for TNBC were tested in varying concentrations (Figure 2 and Figure S1). Proliferation was assessed for 10 different doses over six days of treatment. BCSC lines displayed a strong proliferative heterogeneity in response to chemotherapeutics. BCSC1 and BCSC2 showed a much stronger doxorubicin resistance than BCSC5 (Figure 2A,C,E), while no significant difference was observed in paclitaxel treatment (Figure 2B,D,F). Both BCSC1 and BCSC2 showed more sensitivity to paclitaxel than doxorubicin; in comparison, this phenotype was not observed in BCSC5. Dose response-dependent proliferation was also tested for additional chemotherapeutics (Figure S1). For docetaxel, epirubicin, carboplatin, and cisplatin, BCSC1 showed the strongest resistance, while BCSC5 showed a higher sensitivity than BCSC1 and BCSC2 (Figure S1A–C,D–F,J–L,M–O). For gemcitabine and 5-fluorouracil (5-FU), no significant difference was observed in between the different cell lines (Figure S1G–I,P–R).

### 2.2. Proliferation of BCSCs Was Inhibited after Chemotherapeutic Stress and Recovered Gradually

**Figure 3:**
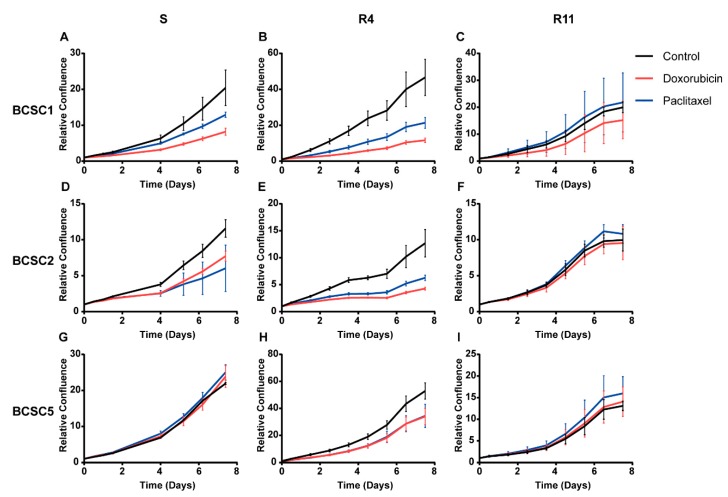
Proliferation of BCSCs was inhibited after chemotherapeutic stress and recovered gradually. BCSCs that survived doxorubicin and paclitaxel treatment were cultured in standard medium to observe their recovery. Surviving cells (S) and recovered cells for 4 days (R4) or 11 days (R11) were harvested and seeded for proliferation analysis. (**A**–**C**) Proliferation analysis for BCSC1 that survived (S) and recovered (R4, R11) from doxorubicin and paclitaxel. (**D**–**F**) Proliferation analysis for surviving and recovered BCSC2. (**G**–**I**) Proliferation analysis for surviving and recovered BCSC5. The *n* = 3, data represent means ± SD.

According to the cancer stem cell hypothesis, this cell type plays an important role in chemoresistance [4,15,17]. We aimed to analyze the potential chemoresistant cell type under stress in more detail. Based on the proliferation data, we determined a sublethal concentration for doxorubicin and paclitaxel individually for each cell line (survival model, Table 1). Treatment with this dosage for six days inhibited cell proliferation significantly, but 20–40% of the cells survived the treatment (surviving chemotherapeutic treatment (S)) (Figure 2). The surviving cancer stem cells were cultured subsequently in standard medium without any chemotherapeutics to mimic the patient situation after chemotherapy. The proliferative capacity of these surviving cells recovered gradually to the state before treatment after 11 days (recover from chemotherapeutics (R4, R11)) (Figure 3).

### 2.3. Chemotherapeutic Stress Induces a Mesenchymal Cell Morphology and Change in EMT Gene Expression, but No Change in Migratory Capacity

**Figure 4:**
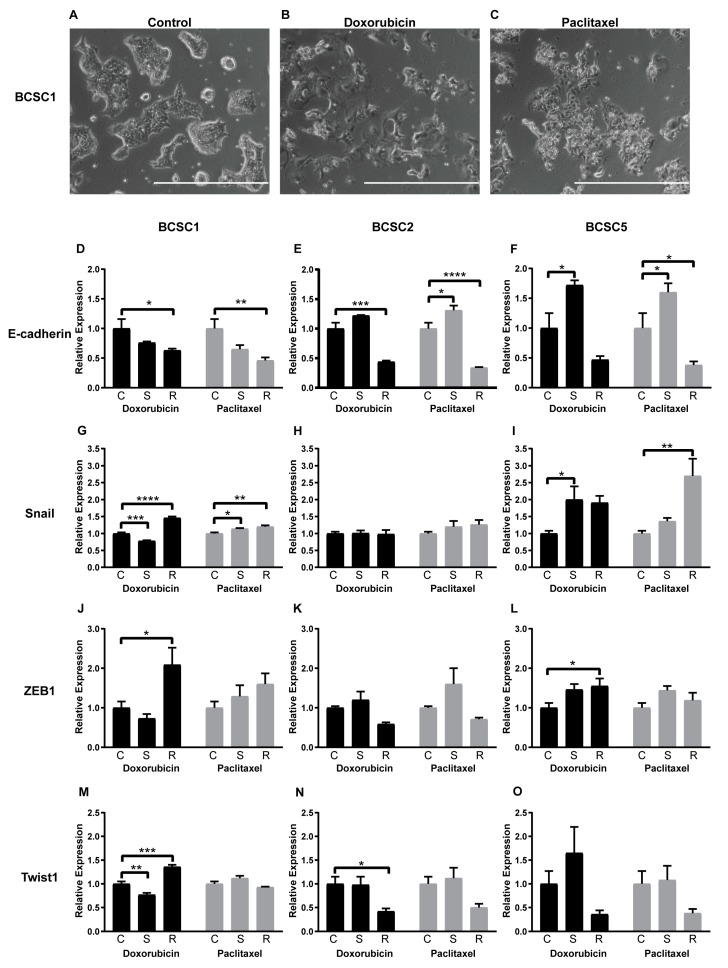
Chemotherapeutic stress changed the cell morphology and the EMT gene expression of the BCSCs at mRNA level. (**A**) Cell morphology of BCSC1. (**B**,**C**) Morphological changes of BCSC1 exposed to doxorubicin (**B**) and paclitaxel (**C**), scale bar 1000 μm. (**D**–**O**) EMT factor expression fold change at mRNA level for surviving (S) and recovered (R) BCSCs from doxorubicin or paclitaxel treatment, compared with untreated control cells (C). The *n* = 3, and data represent means + SEM. * = *p* < 0.05; ** = *p* < 0.01; *** = *p* < 0.001; **** = *p* < 0.0001 by two-way ANOVA.

During chemotherapeutic stress, BCSCs changed morphology quite substantially. BCSC1 is an epithelial cell line, forming well-defined epithelial colonies with cobblestone-like morphology (Figure 4A). After exposure to doxorubicin, cell size increased and cell–cell contacts were reduced (Figure 4B). The cells became elongated and the dense cell clusters disappeared after exposure to both doxorubicin and paclitaxel (Figure 4B,C). Similar morphological changes were observed in BCSC2 and BCSC5 after exposure to doxorubicin and paclitaxel (Figure S2A–F).

In concordance with other research groups [18,19], we recently described an intermediate and dynamic state of EMT in CSCs. In the present study, all examined BCSC lines were in an intermediate state of EMT expressing both mesenchymal and epithelial factors simultaneously (Figure S2G). To determine the intermediate EMT “ground state”, we analyzed expression of Vimentin, ZEB1, Slug, Snail, Twist1, and E-cadherin by quantitative PCR. All factors were detectable but expressed in varying amounts in different BCSC lines. BCSC1 highly expressed E-cadherin, Vimentin, and Slug. BCSC2 showed strong expression of E-cadherin, Vimentin, and Twist1. BCSC5 highly expressed Vimentin and Slug (Figure S2G). This consistently hinted that the intermediate EMT state was upheld by different factors in each individual cell line, likely based on the genetic background of the individual patient. During doxorubicin or paclitaxel treatment and the recovery process, this pattern changed. E-cadherin, the epithelial factor, was downregulated in all recovered BCSCs (R), but upregulated in surviving BCSC2 (S) from paclitaxel and BCSC5 (S) from both treatments (Figure 4D–F). BCSC1, a cell line with epithelial morphology, displayed a low expression state of Snail, ZEB1, and Twist1 (Figure S2G). All mesenchymal factors except Vimentin were upregulated in BCSC1 (R) in recovery from doxorubicin as well as paclitaxel treatment (Figure 4G,J,M and Figure S2H,K). In contrast, BCSC5 showed a strong expression of Vimentin and Slug, but weak expression of Snail and ZEB1 (Figure S2G). During treatments and the recovery process of BCSC5, Twist1, Vimentin and Slug were not regulated significantly (Figure 4O and Figure S2J,M), but upregulations were observed in Snail from paclitaxel (Figure 4I) and ZEB1 from doxorubicin (Figure 4L). BCSC2 had the highest expression level of most EMT factors among the analyzed BCSC lines (Figure S2G). Downregulation of Twist1 in BCSC2 (R) from doxorubicin was the only regulation detected (Figure 4H,K,N and Figure S2I,L). Overall, the EMT induced by chemotherapeutic stress seemed to be driven by different factors in individual cell lines.

It is known that the EMT state strongly impacts the migratory abilities of cells, which might have implications for tumor metastasis [6]. Observing the mesenchymal-like morphological switch and the EMT-related gene expression changes, we therefore performed migration assays using surviving and recovered BCSCs for all three cell lines. No significant differences in the migratory capacity could be detected between surviving, recovered, and untreated control cells (Figure S3).

### 2.4. Chemotherapeutic Stress Enhances the Self-Renewal of the BCSCs

**Figure 5:**
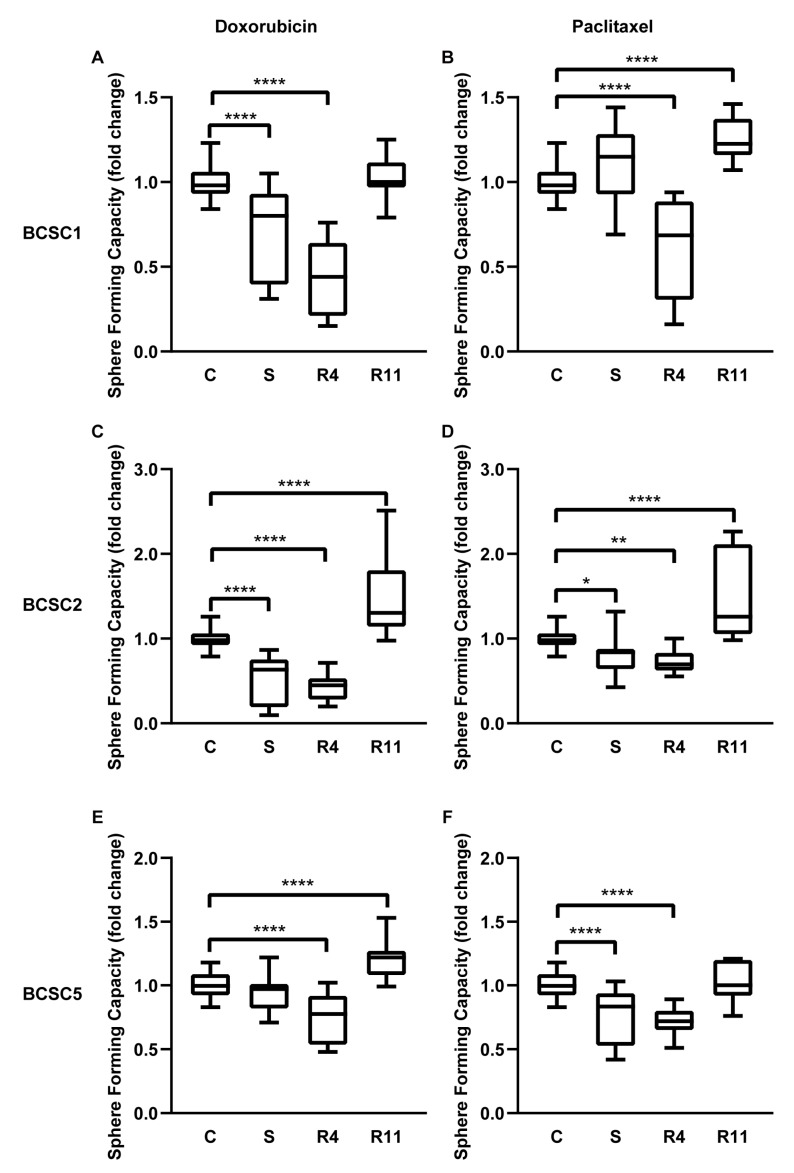
Chemotherapeutic stress enhanced the self-renewal capacity of the BCSCs. (**A**–**F**) Sphere-forming capacity of untreated control BCSCs (C), surviving BCSCs (S), and recovered BCSCs (R4, R11). Data in graph represent relative sphere-forming capacity compared with untreated control cells, *n* = 3. Box plots demonstrate median, lower, and upper quantile range (box lines), and standard deviation range (lines bounded by horizontal lines outside the boxes). * = *p* < 0.05; ** = *p* < 0.01; **** = *p* < 0.0001 by one-way ANOVA.

Concomitant expression of epithelial and mesenchymal factors or an intermediate state of EMT promotes sphere formation, self-renewal, and stemness of cancer stem cells [20]. To analyze the effect of chemotherapeutic stress on the sphere-forming capacity, surviving BCSCs were seeded in 50% Matrigel into 96-well, ultra-low attachment plates. After 10 days, resulting spheres were counted. The sphere-forming capacity was significantly decreased in BCSCs directly after treatment (Figure 5A–F), even more during the first four days of recovery in all BCSCs. Nevertheless, this capacity was significantly increased after 11 days of recovery compared with untreated control cells. Sphere sizes of BCSCs under recovery for 11 days were also analyzed (Figure S4A–C), showing that sphere size was increased in BCSC1 recovered from doxorubicin treatment. Subsequently, we harvested single cells from R11 spheres and evaluated the sphere-forming capacity in a secondary assay. The secondary sphere-forming capacity showed no difference between untreated cells and recovered cells (Figure S4D–F). The effect of increased self-renewal capacity of BCSCs seemed temporary with a delay of 11 days after chemotherapy treatment and lasting no longer than an additional 11 days after the first observation. This could hint at an epigenetic or signaling event causing this observation rather than a permanent selection of a specific cell clone from the BCSC parental culture.

### 2.5. Chemotherapeutic Stress Changes Cancer-Related miRNA Expression Profiles of the BCSCs

In recent years, a growing family of miRNAs was identified as markers as well as potential modulators of chemoresistance [21]. Hence, we analyzed miRNA profile alterations in BCSCs undergoing chemotherapeutic stress. There were 18 potentially relevant miRNAs pre-selected by a comprehensive survey of the current literature related to EMT, chemotherapy, and stemness (Table 2).

**Figure 6:**
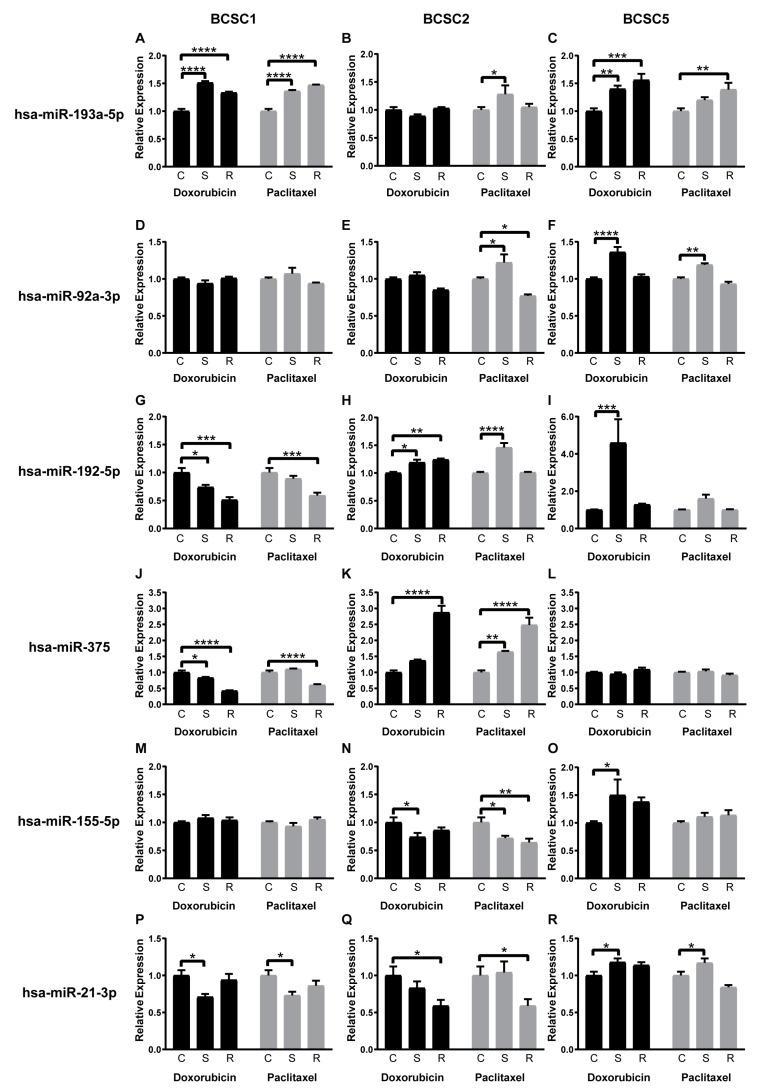
Chemotherapeutic stress-dependent regulation of cancer-related miRNA expression pattern. (**A**–**C**) MiR-193a-5p expression fold change for surviving (S) and recovered (R) BCSCs from doxorubicin or paclitaxel treatments, compared with untreated control cells (C), (**D**–**F**) MiR-92a-3p, (**G**–**I**) MiR-192-5p, (**J**–**L**), MiR-375, (**M**–**O**) MiR-155-5p, (**P**–**R**) MiR-21-3p, *n* = 3, data represent means +SEM. * = *p* < 0.05; ** = *p* < 0.01; *** = *p* < 0.001; **** = *p* < 0.0001 by two-way ANOVA.

Quantitative PCR analysis revealed that the selected microRNAs were reproducibly detectable at heterogeneous levels in all BCSC lines (Figure S5A). In particular, miR-205-5p, which is well known to inhibit ZEB1 expression directly targeting its 3’UTR [66], was inversely expressed compared with ZEB1 in all BCSC lines (Figures S2G and S5A). Throughout the chemotherapeutic stress and recovery processes, most miRNAs in question were differentially regulated (Figure S5B,C). In particular, most of them were upregulated in surviving BCSC5 (S) and recovered BCSC5 (R) subsequently. Likewise, most miRNAs were upregulated in surviving BCSC2 (S) but downregulated in recovered BCSC2 (R). None of the determined miRNAs regulations showed a similar or inversed pattern in the three BCSC lines, but interestingly several parallel expression patterns of six distinct miRNAs could be observed in two respective BCSC lines: The miR-193a-5p showed a similar upregulation pattern between BCSC1 and BCSC5 (Figure 6A–C), while miR-92a-3p displayed a similar regulation between BCSC2 and BCSC5 (Figure 6D–F). The inversed patterns were observed for miR-192-5p and miR-375 between BCSC1 and BCSC2 (Figure 6G–L), for miR-155-5p between BCSC2 and BCSC5 (Figure 6M–O), and for miR-21-3p between BCSC1 and BCSC5 (Figure 6P–R).

## 3. Discussion

Currently, NACT is a commonly accepted treatment strategy for early TNBC [3]. Although it is generally accepted that early stage TNBC is chemosensitive, the high recurrence rates due to fast development of chemoresistance represent the major challenge for this subtype of breast cancer [3]. Cancer stem cells are suggested to be responsible for driving tumor progression, chemoresistance, and metastasis [17]. We were able to isolate and culture BCSCs from patients who received NACT. The response of individual cell lines to chemotherapeutic stress was examined. BCSC1 (representing heavily pretreated cancer stem cells after NACT and further progression under several lines of chemotherapy) showed a much stronger chemoresistance overall than BCSC2 and BCSC5 to most chemotherapeutics, such as doxorubicin, epirubicin, docetaxel, and cisplatin. One could hypothesize from this finding that a stronger exposure to chemotherapy, as it is seen in the clinical routine for the natural course of disease for TNBC, leads to the accumulation of chemoresistance in BCSCs against distinct cytotoxic agents. Therefore, BCSC1 reflects the acquired chemoresistance phenotype in TNBC. This observation needs to be evaluated on a much bigger patient cell line cohort to be substantiated.

Since chemoresistance of TNBC is multifaceted, based on the interplay of drug efflux [67], apoptosis [68,69], cancer stem cell phenotype [17], tumor microenvironment [70], and multiple signaling pathways [71], the mechanism of chemoresistance in TNBC has to be analyzed under many different aspects. In the present study, characteristics of BCSCs under chemotherapeutic stress were analyzed. Therefore, we set up a survival model to mimic the tumor cells under and after chemotherapeutic stress in the in vivo situation. Doxorubicin and paclitaxel, which are the most commonly used agents for TNBC patients, were selected for further analysis. Since it is almost impossible to retrace the amount and concentration of a chemotherapy agent that actually reaches BCSCs in patients [72], we determined a drug-specific sublethal concentration using the dynamic data from proliferation assays [73]. Surviving and recovered cells were analyzed in the present analyses.

We found epithelial and mesenchymal genes co-expressed in BCSCs. This plasticity of BCSCs linked to an intermediate EMT state could be advantageous for metastasis and stemness [5,74]. In our present study, chemotherapeutic stress on BCSCs elicited an obvious morphological change towards a more mesenchymal phenotype. The EMT process is marked by morphological and genetic changes, in particular a dramatic change of EMT-related factors [75]. Loss of epithelial factors is considered to be a fundamental event in EMT, which is accompanied by upregulation of mesenchymal factors, such as Vimentin, ZEB1, Slug, Snail, and Twist1 [76]. Here, E-cadherin was downregulated in almost all the recovered BCSCs. In contrast, the mesenchymal factor regulation was very heterogeneous. Snail, ZEB1, and Twist1 were upregulated in BCSC1, while Snail and Twist1 were upregulated in BCSC5. Minor changes of EMT-related factors in BCSC2 may be due to the high expression of EMT factors in “ground state” (Figure S2G). Together these findings imply that EMT regulation is a common reaction in CSCs upon chemotherapeutic stress. Nevertheless, differentially regulated EMT factors may be attributed to genetic and epigenetic differences between individual CSC cultures and consecutively distinct patients. Although in the conventional model of metastasis, EMT plays an important role in promoting metastasis by inducing migration and invasion [77,78,79], recent evidence showed that increased migration is not an inexorable consequence of EMT regulation [80,81]. Cai et al. and Shamir et al. demonstrated that downregulation of E-cadherin could completely block the migratory process [82,83]. We made a similar observation of changed cell morphology and EMT gene expression but not migratory capacity upon chemotherapeutic stress. A BCSC tail vein injection [84] will be performed in the future to confirm the potentially metastatic capacity of these surviving and recovered BCSCs.

Morel et al. showed that primary human mammary epithelial cells (HMECs) with a CD44^−^/CD24^+^ phenotype can generate tumorigenic CD44^+^/CD24^−^ cells after transformation with oncogenes and cancer-associated genes. Aberrant activation of the Ras/MAPK signaling pathway led to this phenotype, suggesting CSCs can be derived by EMT [74]. Most of our BCSC cultures have a CD44^+^/CD24^−^ phenotype even without chemotherapeutic stress [16]. Nevertheless, we observed an upregulated self-renewal capacity phenotype from primary BCSCs under chemotherapeutic stress. This phenotype could be the result of clonal selection or chemotherapeutic stress-induced regulation. In our analyses the phenotype was observed in all BCSCs which underwent chemotherapeutic stress and was only temporary. Therefore, we inferred that it was more likely a regulation rather than selection. The stemness induced by EMT was reported to be regulated in conventional cell lines by various genes, such as ZEB1, Twist1, Slug, Snail, and Vimentin [5,74,85]. We saw a similar pattern where the EMT was regulated by different factors in the individual lines, but we observed a temporarily upregulated self-renewal capacity under chemotherapeutic stress in primary BCSCs (Figure 5). As a gold standard assay which confirms the capacity of self-renewal and propagation [86], orthotopic transplantation into immunocompromised mice will be performed in the future.

The function of miRNAs during tumor growth and progression as well modulation of chemoresistance is diverse and complex. MiR-107 could induce or inhibit metastasis in different cancer types [24,25], while miR-200a-3p could influence different signaling pathways in hepatocellular carcinoma [29,30]. In the present study, we observed a similar or inversed regulation of six miRNAs in just two of the three BCSC lines considered. MiR-193a-5p was proved to inhibit metastasis and proliferation in non-small cell lung cancers (NSCLCs) and breast cancers [54,55]. The upregulation of miR-193a-5p had the same pattern with Snail and ZEB1 in BCSC1 and BCSC5, which could result in the inhibition of migration in BCSCs with upregulation of mesenchymal factors. Xu et al. reported that upregulation of miR-92a-3p led to reduced proliferation and colony formation in colorectal cancer [59]. Combining the regulation of miR-92a-3p expression and sphere-forming capacity, miR-92a-3p seemed to play an important role in the self-renewal of BCSCs. MiR-375 was reported to inhibit EMT in breast cancer while inducing docetaxel resistance in prostate cancer [47,50]. MiR-192-5p was significantly abolished in hepatocellular carcinomas expressing high levels of CSC markers, but enhanced doxorubicin sensitivity in breast cancer [51,52]. MiR-21-3p played an important role in mediating cisplatin resistance in ovarian cancer and reduced TRAIL-mediated apoptosis in liver cancer stem cell [34,35]. Mir-155-5p suppressed EMT in breast cancer but enhanced metastasis in oral squamous cell carcinoma [27,28]. In our present study, miR-375, miR-192-5p, miR-21-3p, and miR-155-5p showed a completely inversed regulation in two BCSC lines under chemotherapeutic stress. All these findings implied a heterogeneous effect on miRNA regulation in individual BCSC lines under chemotherapeutic stress. The pathway of these miRNAs requires further research in the future.

In summary, we constructed a survival model using BCSCs to mimic tumor cells in patients under chemotherapy. During the survival and recovery process, an EMT was observed, which did not induce proliferation and migration, but in contrast increased BCSC self-renewal. This process is regulated by various miRNAs and EMT-related signaling pathways in individual BCSC lines.

## 4. Materials and Methods

### 4.1. Cell Culture

BCSC1 and BCSC2 were isolated in 2014 from primary triple-negative breast tumors of patients who had received NACT, as shown in our previous work [16]. Patient of BCSC5 was operated on at the Department of Obstetrics and Gynecology at the University Medical Centre Freiburg in 2014. Tumor tissue specimens for BCSC isolation and paraffin embedding were obtained from pathologists of the tumor bank of the Comprehensive Cancer Centre Freiburg. Written informed consent was obtained from the patients. All experiments were performed in accordance with the Declaration of Helsinki. We confirm that all experimental protocols were approved by the Institutional Review Board in the Ethics vote 307/13 (independent Ethics Committee University of Freiburg). BCSCs were cultured in a 2D environment, with MSC medium at 37 °C under low oxygen conditions (3% O_2_, 5% CO_2_, 92% N_2_). For the subculture, the cells were first washed with DPBS (Gibco, 14200-067, Paisley, UK) and detached with accutase (Sigma-Aldrich, A6964, St. Louis, MO, USA) at 37 °C for ~20 min. Mammary epithelial basal medium (MEBM, Lonza, CC-3151, Verviers, Belgium) medium was added with the same volume to dilute the accutase. Following centrifugation at 200× g for 3 min, the supernatant was discarded. The cell pellet was washed with 1 mL MEBM and centrifuged again at 200× g for 3 min. The obtained cell pellet was suspended in MSC medium. The 15 × 10^4^ cells were seeded in 1 mL MSC medium containing 2% Matrigel (ice-cold, Corning, 354230, Bedford, MA, USA) in a 6-cm culture dish. After solidification of the Matrigel at 37 °C for 30 min, the dish was topped up with 2 mL of MSC medium. Medium was changed every 3 days. Cells were split once a week. All BCSC lines were authenticated on a six-month basis by the high-throughput multiplex human cell authentification test (MCA) developed at the DKFZ in 2016 [87]. Mycoplasma tests were conducted every 3 months via PCR detection kit of “Venor^®^GeM Advance” (Minerva Biolabs, 11-7048, Berlin, Germany). Experiments with cells were conducted in a passage lower than 35.

### 4.2. MSC Medium

The mammary stem cell (MSC) medium was composed of MEBM, supplemented with 1× B27 (Gibco, 17504-044, Grand Island, NY, USA), 1× amphotericin B (Gibco, 15290-026, Grand Island, NY, USA), and 1× penicillin–streptomycin (Gibco, 15140-122, Grand Island, NY, USA). Furthermore, epidermal growth factor (20 ng/mL, PeproTech, AF-100-15, Rocky Hill, NJ, USA), heparin (4 μg/mL, Sigma-Aldrich, H3393-25KU, Taufkirchen, Germany), fibroblast growth factor (20 ng/mL, PeproTech, AF-100-18B, Rocky Hill, NJ, USA), gentamicin (35 μg/mL, Gibco, 15750-045, Paisley, UK), and rho kinase inhibitor (500 nmol/L, Calbiochem, 555552, Darmstadt, Germany) were added.

### 4.3. Chemotherapy Agent

Doxorubicin (Doxorubicin Accord^®^, Freilassing, Germany, 2 mg/mL), docetaxel (Docetaxel Accord^®^, Freilassing, Germany, 20 mg/mL), and 5-fluorouracil (Fluorouracil Accord^®^, Freilassing, Germany, 50 mg/mL) were purchased from Accord. Epirubicin (Epimedac^®^, Wedel, Germany, 2 mg/mL) and carboplatin (Carbomedac^®^, Wedel, Germany, 10 mg/mL) were purchased from Medac. Paclitaxel (NeoTaxan^®^, Holzkirchen, Germany, 6 mg/mL) and gemcitabine (Gemcitabin Hexal^®^, Holzkirchen, Germany, 40 mg/mL) were purchased from Hexal. Cisplatin (Cisplatin Teva^®^, Ulm, Germany, 1 mg/mL) was purchased from Teva. All agents were dissolved in physiological solution.

### 4.4. Flow Cytometry

Cells were detached and counted as described above. To analyze the expression of surface markers, approximately 1 × 10^5^ cells were washed with staining buffer (DPBS + 1% bovine serum albumin (BSA, Carl Roth, 8076.2, Karlsruhe, Germany)) and stained for 20 min at room temperature in the dark with the antibodies diluted in staining buffer as below: Anti-CD24 (eBioscience, Bleiswijk, Netherlands, 46-0247; 1:100), anti-CD44 (eBioscience, Bleiswijk, Netherlands, 12-0441-81; 1:1000), anti-EpCAM (eBioscience, Bleiswijk, Netherlands, 660 50-9326; 1:100), and anti-CD49f (eBioscience, Bleiswijk, Netherlands, 46-0495; 1:200). Cells were analyzed using BD LSRFortessa^TM^ cell analyzer (BD, San Jose, CA, USA) and FlowJo software (Version 6, Ashland, OR, USA).

### 4.5. Orthotopic Breast Cancer Xenografts

All animal studies and experiments were performed in accordance with German Animal Welfare regulations and in accordance with an Institutional Animal Care and Use Committee as described in the animal protocol G13/114. Indicated amounts of BCSC5 were mixed with 1 × 10^6^ irradiated NuFF fibroblasts (newborn human foreskin fibroblasts, p11, GlobalStem, GSC-3002, Rockville, MD, USA) and suspended in a 1:1 mixture of MSC medium and Matrigel in a total volume of 40 μL per gland. At 4–5 weeks old, NOD/SCID females were anesthetized using an isoflurane inhalator. A small sagittal incision (no longer than 1.0 cm) on the shaved and sterilized abdomen allowed access to both the no. 4 mammary gland. The cell suspension was injected into the mammary fat pad of the no. 4 gland on both sides of the animal. Each transplant was localized distal to the lymph node in the gland. Surgical incisions were sealed by suturing with a 5/0 thread (Ethicon, Z995, New Brunswick, NJ, USA). Animals were monitored twice weekly for weight and tumor growth, which was determined by caliper measurement. Tumor volumes were calculated using the formula 4/3 × π × r3.

### 4.6. Immunohistochemistry

Tissue specimens were fixed in 10% formalin and subsequently embedded in paraffin. Then, 2-μm-thick paraffin-embedded tissue sections were mounted onto glass slides. All slides were stored for two days at 58 °C in a drying chamber, subsequently deparaffinized using xylene and hydrated with ethanol. Human and corresponding xenograft tumor tissues were stained using the following antibodies: Anti-ER (clone EP1, code IR084, Dako, Santa Clara, CA, USA), anti-PR (clone PgR 636, code IR068, Dako, Santa Clara, CA, USA), anti-HER2 (code A0485, Dako, Santa Clara, CA, USA). Counterstaining was performed with hemalum before adding a coverslip. As positive controls, a patient-derived physiological mammary gland was used for ER and PR staining. For HER2, tissue specimens from HER2-positive breast cancer patients (score 3) [88] were included. TNBC was defined as ER-, PR-, and HER2-negative (score < 2) [89].

### 4.7. Cell Proliferation and Time–Dose Response Assay

Cells were detached and counted. For proliferation assays, 96-well plates (Corning, 353072, Durham, NC, USA) were coated with 50 µL of MSC medium containing 2% of Matrigel. After incubation at 37 °C for 30 min to solidify the Matrigel, 4 × 10^3^ single cells were seeded in 50 µL MSC medium per well. Cell confluence was subsequently monitored for 6 days by taking images every 6 h using the IncuCyte^®^ S3 Live-Cell Analysis System (Sartorius, Ann Arbor, MI, USA).

For the time–dose response assays, the wells of a black, 384-well plate (Greiner, 781091, Kremsmünster, Austria) were coated with 10 μL of MSC medium containing 2% of Matrigel. After incubation at 37 °C for 30 min to solidify the Matrigel, 1 × 10^3^ single cells were seeded in 30 µL MSC medium per well and grew for 24 h. Afterwards, the chemotherapeutics of interest were added at 10 different concentrations ranging from 3 pg/mL to 100 ng/mL for docetaxel, 30 pg/mL to 1 μg/mL for doxorubicin, paclitaxel, and epirubicin, 100 pg/mL to 3 μg/mL for gemcitabine, 1 ng/mL to 30 μg/mL for cisplatin, 3 ng/mL to 100 μg/mL for 5-fluorouracil, 10 ng/mL to 300 μg/mL for carboplatin. Cell confluence was subsequently monitored for 6 days by taking images every 3 h using the IncuCyte^®^ S3 Live-Cell Analysis System. Both assays were analyzed by IncuCyte^®^ system with percentage confluence from each image.

### 4.8. Survival Model Construction

To mimic the in vivo environment of tumor cells under and after chemotherapy, a survival model was constructed using BCSCs. After chemotherapeutics treatment at a specific dosage, the small cluster of BCSCs that survived was subsequently cultured in medium without chemotherapeutics, in order to mimic the patient situation after chemotherapy, to see if their proliferative capacity could recover. Although a short-term treatment at higher concentration could lead to the same or an even better proliferative inhibition, the proliferative capacity of these surviving cells hardly recovered subsequently. Finally, based on the dynamic data from time–dose response assays described above, we determined the sublethal concentration for doxorubicin and paclitaxel in the survival model (Table 1). Treatment with those defined dosages for six days inhibited cell proliferation significantly, but 20–40% of cells survived the treatment. After 11 days their proliferative capacity recovered gradually to the state before treatment, i.e., when these cells were cultured in standard medium without any chemotherapeutics after treatment.

### 4.9. RNA Extraction

Total RNAs from the cells were isolated by using the “miRNeasy^®^ Mini Kit” (Qiagen, 1038703, Hilden, Germany) according to the manufacturer’s instructions. Isolated RNA was quantitatively determined using the NanoDrop One (ThermoFisher, Madison, WI, USA). RNA samples were stored at −80 °C until further processing.

### 4.10. Reverse Transcription and Quantitative PCR

Reverse transcription was performed on the mRNA using the EvoScript Reverse Transcriptase (Roche, 07912323001, Mannheim, Germany) and Primer Random (Sigma-Aldrich, 11034731001, Mannheim, Germany). The reaction was carried out at 45 °C for 15 min and 85 °C for 5 min, and stopped by 65 °C for 15 min. Processed complementary DNA was stored at −20 °C. For gene expression, quantitative PCR was performed using Universal Probe Library (UPL Roche, Mannheim, Germany) system by Roche LightCycler480. ACTB was used as housekeeping gene. Primers and probes are shown in Table 3.

For miRNA, reverse transcription was performed using the “TaqMan^®^ Advanced miRNA cDNA Synthesis Kit” (Themofisher, A28007, Carlsbad, CA, USA) according to the supplier’s protocol. 10 ng total RNA plus 10 pM ath-miR159a as spike-in control were used per reaction. Processed cDNA was stored at −20 °C. Potentially relevant miRNAs were determined by a comprehensive survey of the current literature applying the PubMed interface. There were 18 miRNAs related to EMT, chemotherapy, and stemness in various types of cancer selected (Table 2). Quantitative PCR for expression of miRNA was performed using TaqMan^®^ Advanced MicroRNA Assay (Thermofisher, Pleasanton, CA, USA) according to the manufacturer’s instructions.

### 4.11. Migration Assay

To analyze cell migratory capacity, 3 × 10^4^ cells were seeded in 96-well plates (Corning, 353072, Durham, NC, USA). After two days of culture, once confluence was reached, scratch wounds were inflicted using the IncuCyte^®^ WoundMaker (Sartorius, Ann Arbor, MI, USA) according to the manufacturer’s instructions. Scratch wound closure was subsequently monitored for 48 h by taking images every 2 h using the IncuCyte^®^ S3 Live-Cell Analysis System. Relative wound density (RWD) was used to quantify the capacity of migration.

### 4.12. Sphere-Forming Assay and Anchorage-Independent Growth Assay

To quantify the sphere-forming capacity of BCSCs, cells were seeded as 5 replicates in 50% Matrigel into 96-well, ultra-low attachment plates (Corning, 3474, Kennebunk, ME, USA). After 10 days, spheres with a diameter of more than 50 µm were counted. Sphere-forming capacity was calculated based on the number of initially seeded cells. Photos of spheres were taken using Invitrogen™ EVOS™ FL Auto Imaging System (ThermoFisher, Bothell, WA, USA). Sphere size was analyzed using ImageJ software (NIH, Bethesda, MD, USA). For quantification of anchorage-independent sphere-forming capacity, 1 × 10^3^ single cells were seeded into 96-well, ultra-low attachment plates in MSC medium containing 1% methylcellulose (Sigma, M0512, Taufkirchen, Germany). After 7 days, spheres with a diameter of more than 50 μm were counted.

To evaluate a secondary sphere-forming capacity, spheres were harvested 11 days after seeding. Cells were split using Dispase (Corning, 354235, Bedford, MA, USA) to solve residual Matrigel and accutase for sphere dissociation. Afterwards, 200 single cells were seeded as 5 replicates in 50% Matrigel into 96-well, ultra-low attachment plates and spheres were counted after 10 days as described above.

### 4.13. Statistics

Significance was calculated by a one-way ANOVA or two-way ANOVA as indicated with GraphPad Prism (V8.0.2). Data represents means ± SD or means ± SEM as indicated; not significant = *p* ≥ 0.05; * = *p* < 0.05; ** = *p* < 0.01; *** = *p* < 0.001; **** = *p* < 0.0001.

## Figures and Tables

**Table 1:** Concentrations used in the survival model.

ID	Doxorubicin	Paclitaxel
BCSC1	30 ng/mL	3 ng/mL
BCSC2	30 ng/mL	3 ng/mL
BCSC5	3 ng/mL	1 ng/mL

**Table 2:** Selected miRNA profile related to EMT, chemotherapy, and stemness in cancers.

miRNA	Cancer Types Reported Relevant to EMT, Chemotherapy and Stemness	Reference
hsa-miR-107	NCLC, glioma, gastric cancer, breast cancer.	[22,23,24,25]
hsa-miR-155-5p	Colorectal cancer, breast cancer, oral squamous cell carcinoma.	[26,27,28]
hsa-miR-200a-3p	Hepatocellular carcinoma.	[29,30]
hsa-miR-205-5p	Breast cancer, colon cancer, ovarian cancer.	[31,32,33]
hsa-miR-21-3p	Liver cancer, ovarian cancer.	[34,35]
hsa-miR-21-5p	Colon cancer, NCLC.	[36,37]
hsa-miR-23b-3p	Gastric cancer, hepatocellular carcinoma, acute myeloid leukemia.	[38,39,40]
hsa-miR-429	Breast cancer, ovarian cancer.	[41,42]
hsa-miR-125b-5p	Gallbladder cancer, ovarian cancer.	[43,44]
hsa-miR-194-5p	Colorectal adenocarcinoma, ovarian cancer.	[45,46]
hsa-miR-375	Breast cancer, cervical cancer, prostate cancer.	[47,48,49,50]
hsa-miR-192-5p	Hepatocellular carcinoma, breast cancer.	[51,52]
hsa-miR-193a-5p	Prostate cancer, NCLC, breast cancer.	[53,54,55]
hsa-miR-193b-3p	Cervical cancer, T-cell acute lymphoblastic leukemia.	[56,57]
hsa-miR-92a-3p	Glioma, colorectal cancer.	[58,59]
hsa-miR-361-5p	Glioma, gastric cancer.	[60,61]
hsa-miR-30a-5p	Ovarian Cancer, melanoma.	[62,63]
hsa-miR-100-5p	Breast cancer, lung cancer.	[64,65]

**Table 3:** Sequences of polymerase chain reaction primers.

Genes	Forward Primer	Reverse Primer	Probes
ACTB	CCAACCGCGAGAAGATGA	CCAGAGGCGTACAGGGATAG	No. 64
E-cadherin	CCCGGGACAACGTTTATTAC	GCTGGCTCAAGTCAAAGTCC	No. 35
Vimentin	GACCAGCTAACCAACGACAAA	GAAGCATCTCCTCCTGCAAT	No. 39
ZEB1	AACTGCTGGGAGGATGACAC	TCCTGCTTCATCTGCCTGA	No. 57
Slug	TGGTTGCTTCAAGGACACAT	GCAAATGCTCTGTTGCAGTG	No. 7
Snail	GCTGCAGGACTCTAATCCAGA	ATCTCCGGAGGTGGGATG	No. 11
Twist1	AGCTACGCCTTCTCGGTCT	CCTTCTCTGGAAACAATGACATC	No. 58

## Supplementary Materials

Supplementary materials can be found at https://www.mdpi.com/1422-0067/21/2/404/s1.

## Abbreviations

5-FU	5-Fluorouracil
BCSC	Breast cancer stem cell
EMT	Epithelial-mesenchymal transition
ER	Estrogen receptor
FAC	5-Fluorouracil, doxorubicin, and cyclophosphamide
FEC	5-Fluorouracil, epirubicin, and cyclophosphamide
HER2	Human epidermal growth factor receptor 2
HMEC	Human mammary epithelial cell
MEBM	Mammary epithelial basal medium
miRNA	microRNA
NACT	Neoadjuvant chemotherapy
NSCLC	Non-small cell lung cancer
PR	Progesterone receptor
RWD	Relative wound density
TAC	Docetaxel, doxorubicin, and cyclophosphamide
TC	Docetaxel and cyclophosphamide
TNBC	Triple-negative breast cancer
