# Supplementary material for: Chemotherapeutic Stress Influences Epithelial–Mesenchymal Transition and Stemness in Cancer Stem Cells of Triple-Negative Breast Cancer
Source: Int J Mol Sci. 2020 Jan 8;21(2):404. doi: 10.3390/ijms21020404 (PMC7014166; doi:10.3390/ijms21020404)
Supplement: Supplementary file 1 [file ijms-21-00404-s001.pdf]

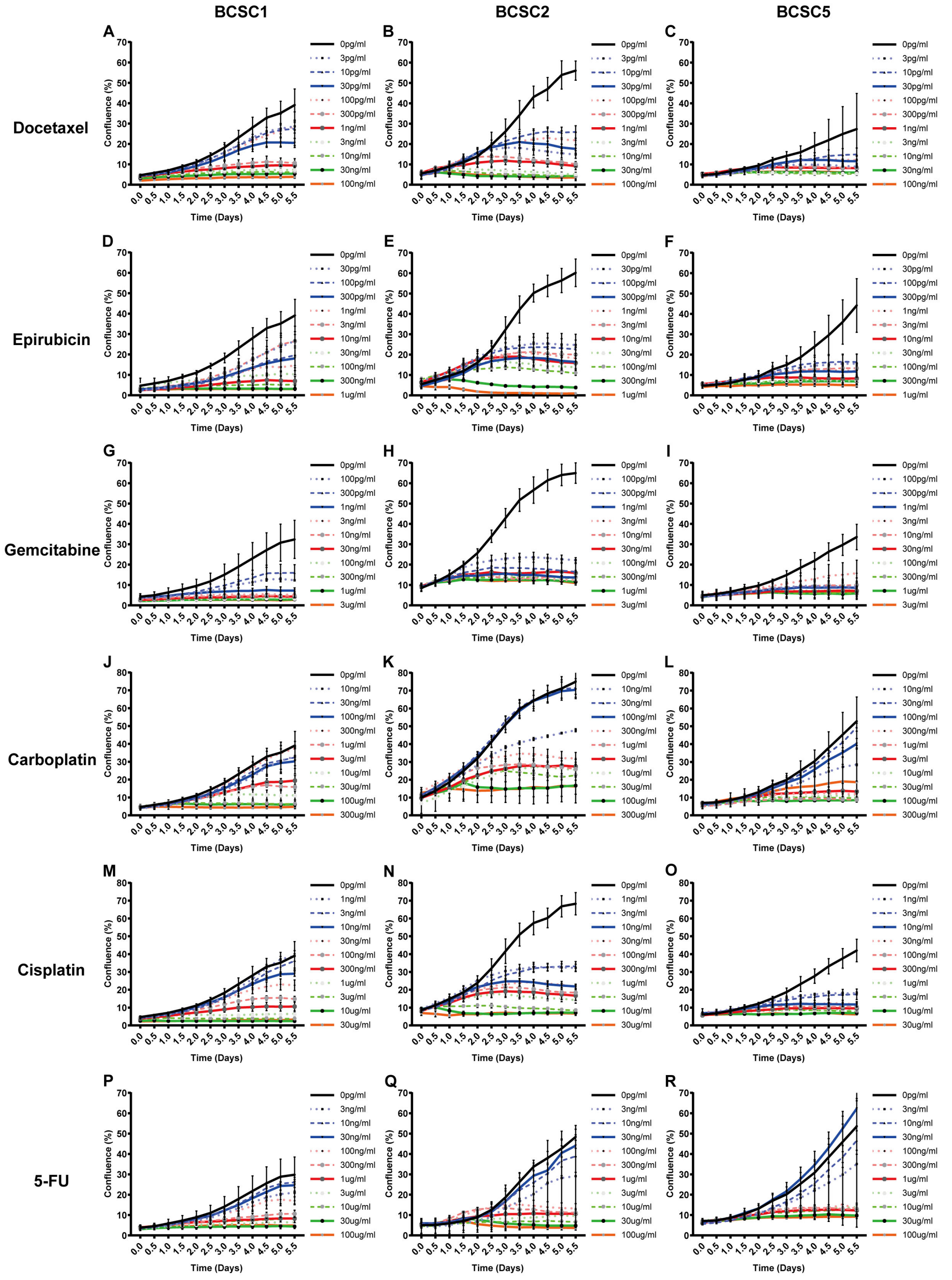

Figure S1. BCSCs from TNBC show different resistance against common chemotherapeutics.

BCSCs were treated with common chemotherapeutics at 10 different concentrations. Cell confluence was recorded and analyzed. (**A**–**C**) Docetaxel. (**D**–**F**) Epirubicin. (**G**–**I**) Gemcitabine. (**J**–**L**) Carboplatin. (**M**–**O**) Cisplatin. (**P**–**R**) 5-FU.

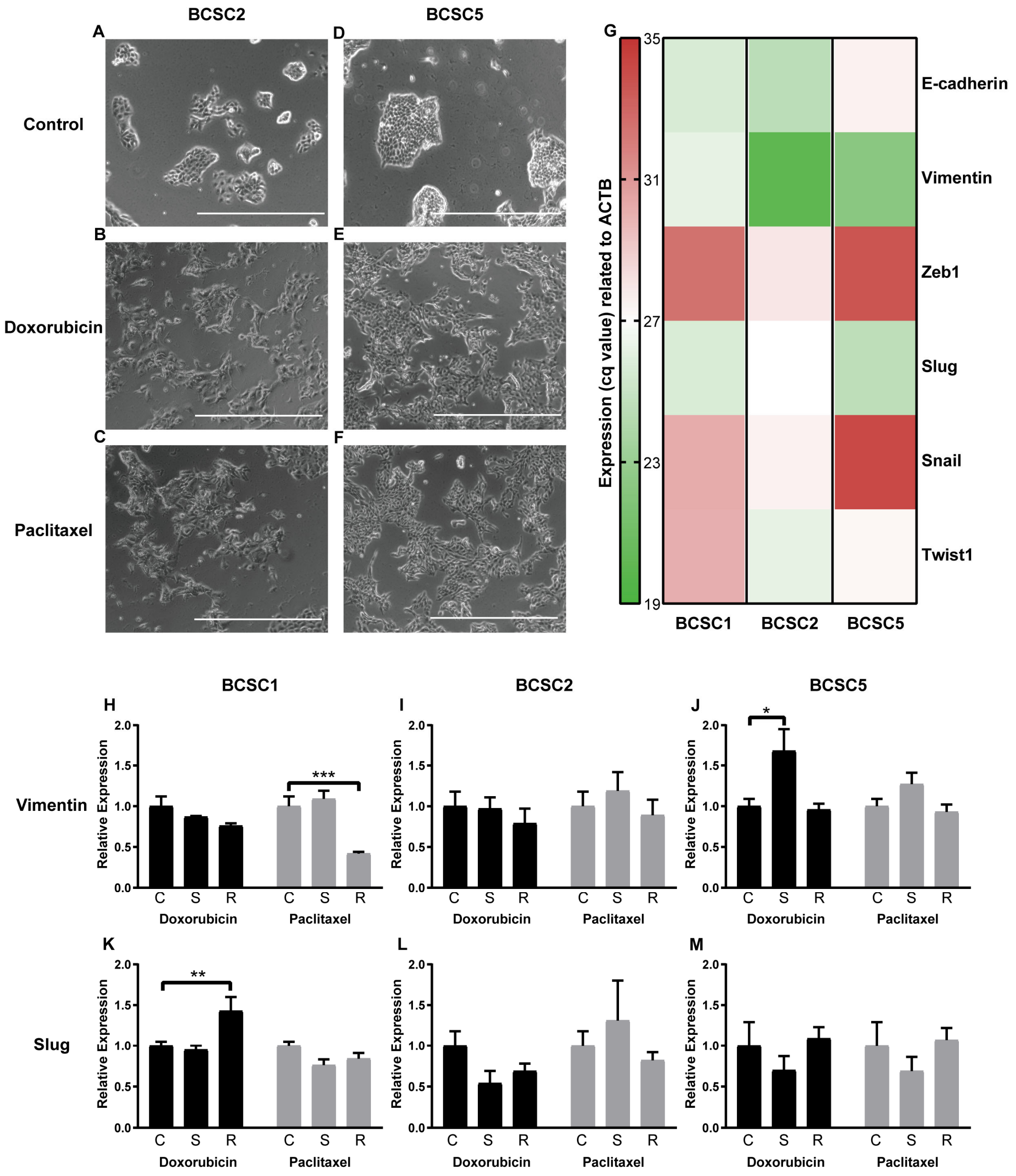

Figure S2. Chemotherapeutic stress changed the cell morphology and the EMT gene expression of the BCSCs at mRNA level.

(**A**) Cell morphology of BCSC2. (**B**–**C**) Morphological changes of BCSC2 exposed to doxorubicin (**B**) and paclitaxel (**C**). (**D**) Cell morphology of BCSC5. (**E**–**F**) Morphological changes of BCSC5 exposed to doxorubicin (**E**) and paclitaxel (**F**), scale bar 1000 μm. (**G**) Heatmap represents the absolute EMT gene expression corrected by ACTB from Cq values obtained from quantitative PCR. Green is representing high expression while red is representing low expression. Depending on this genetic background, BCSCs showed an intermediate state of EMT. (**H**-**M**) EMT factor expression fold change at mRNA level for BCSCs that survived (S) and recovered (R)

from chemotherapeutic stress compared with untreated control cells (C). The $n = 3$, and data represent means + SEM. $* = p < 0.05$; $** = p < 0.01$; $*** = p < 0.001$ by two-way ANOVA.

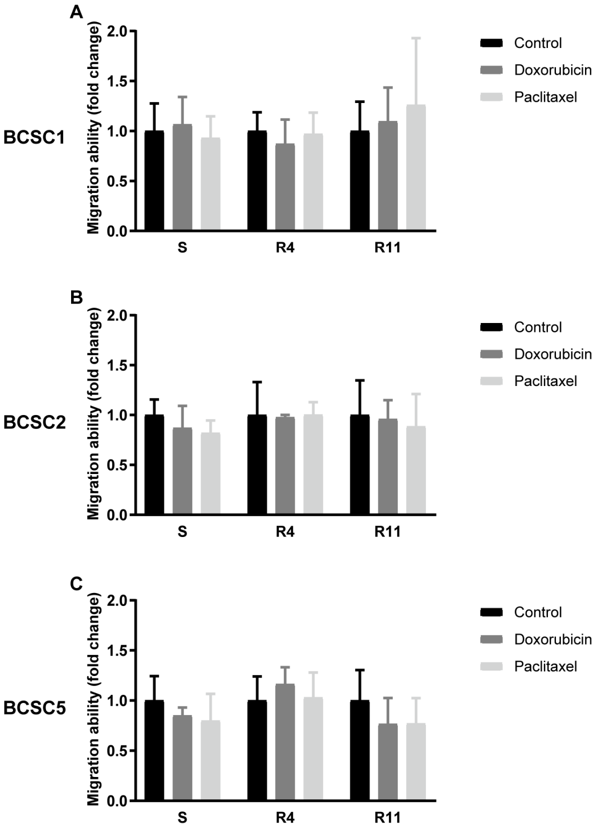

Figure S3. Chemotherapeutic stress did not change the migratory capacity of BCSCs.

(**A**) Migration assays for surviving and recovered BCSC1. (**B**) Migration assays for surviving and recovered BCSC2. (**C**) Migration assays for surviving and recovered BCSC5. BCSCs that survived the chemotherapeutic stress were seeded in 96 well plates to perform migration assays using the IncuCyte® System. Relative wound density (RWD) was used to analyze the migratory capacity. All BCSCs that survived (S) and recovered (R4, R11) from either doxorubicin or paclitaxel showed no significant difference in migratory capacity compared with untreated control cells (C). The $n = 3$, and data represent means + SD.

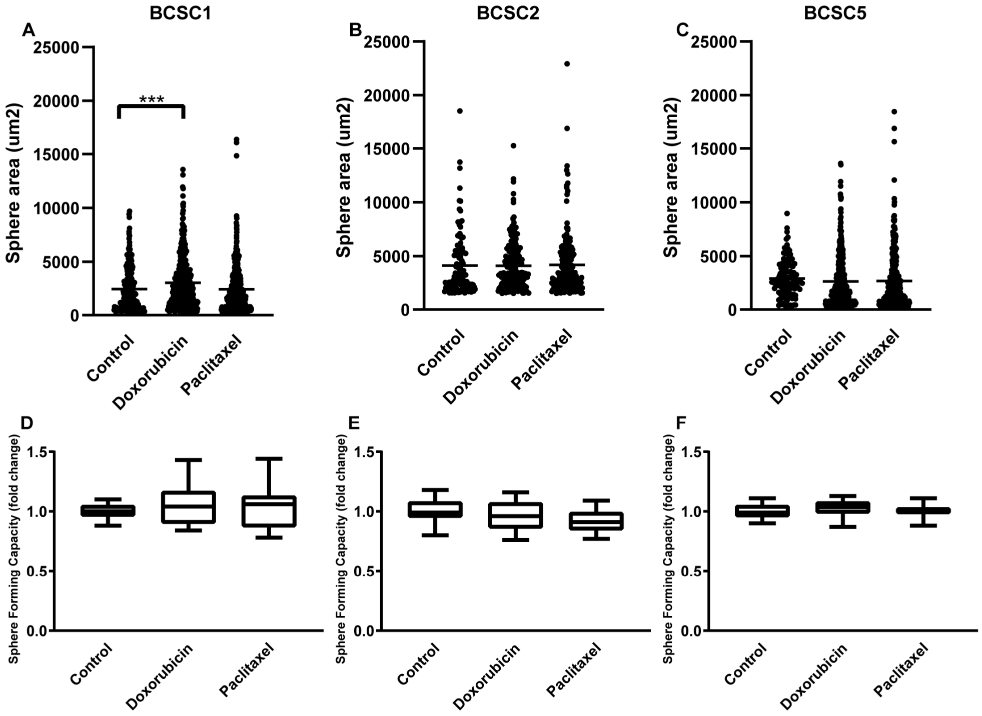

Figure S4. Sphere size and secondary sphere forming capacity in surviving BCSC recovered for 11 days. (**A**–**C**) Individual values of sphere size (μm^2) from surviving BCSCs recovered in standard culture for 11 days. Black lines represent means. (**D**–**F**) Secondary sphere forming capacity of recovered BCSCs (R11). Data in graph represents relative sphere forming capacity compared with untreated control cells. The n = 3. Box plots demonstrate median, lower and upper quantile range (box lines), and standard deviation range (lines bounded by horizontal lines outside the boxes).

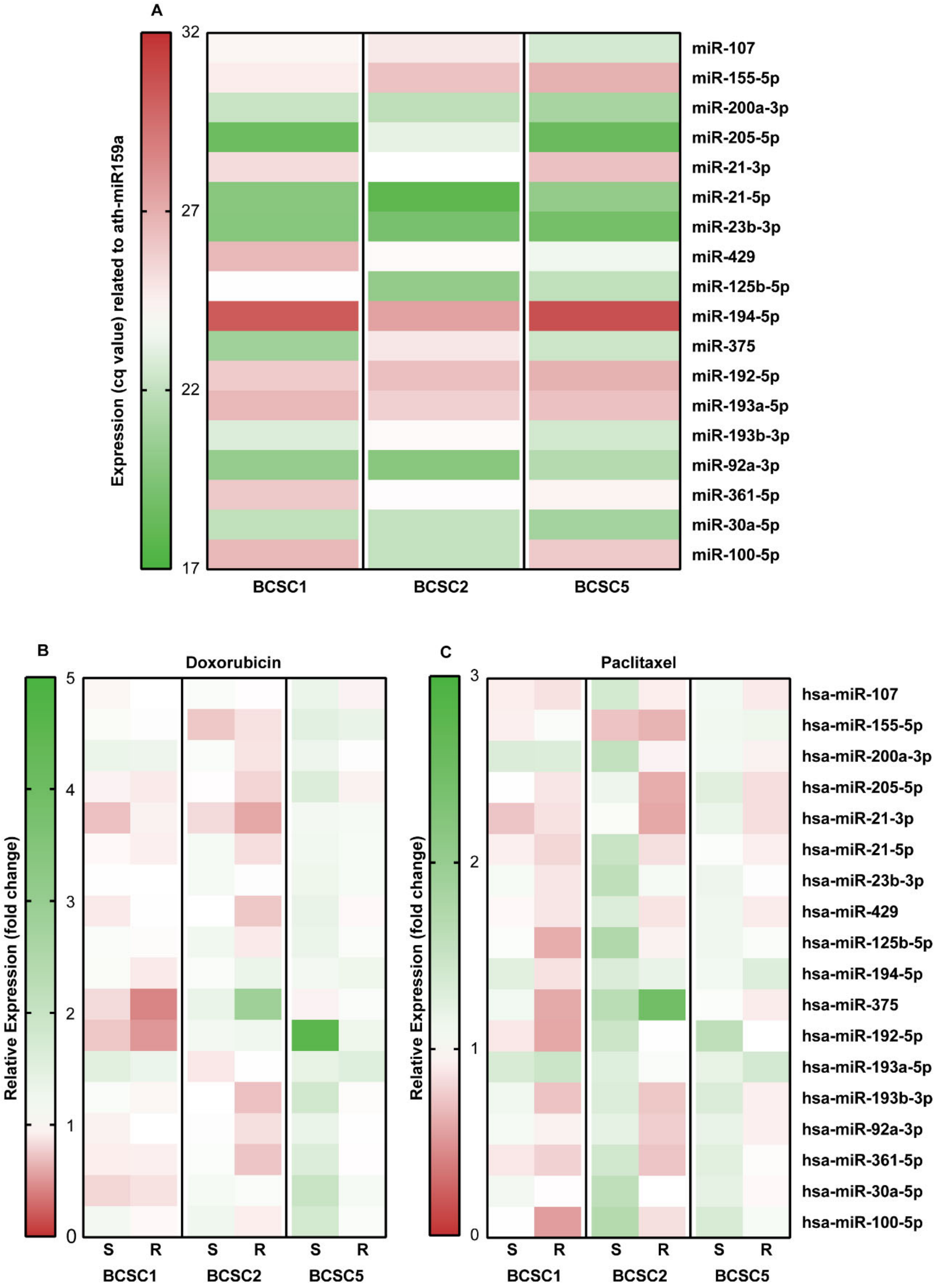

Figure S5. Cancer related miRNA expression profiles in BCSCs under chemotherapeutic stress.

(**A**) Heatmap represents the cancer related miRNA expression level. Data in heatmap represents the absolute expression corrected by ath-miR159a from Cq value obtained from quantitative PCR. Green is representing high expression while red is representing low expression. (**B**–**C**) Heatmap represents the cancer related miRNA

regulations of BCSCs survived (S) and recovered (R) from doxorubicin (**B**) and paclitaxel (**C**). Data in heatmap represents relative expression compared with untreated control cells. Green is representing upregulation while red is representing downregulation. The $n = 3$, and data represent means of fold change.
